# Moderate Exercise Promotes Human RBC-NOS Activity, NO Production and Deformability through Akt Kinase Pathway

**DOI:** 10.1371/journal.pone.0045982

**Published:** 2012-09-25

**Authors:** Frank Suhr, Julian Brenig, Rebecca Müller, Hilke Behrens, Wilhelm Bloch, Marijke Grau

**Affiliations:** 1 Department of Molecular and Cellular Sports Medicine, German Sport University Cologne, Cologne, Germany; 2 The German Research Center of Elite Sport, German Sport University Cologne, Cologne, Germany; Goethe University, Germany

## Abstract

**Background:**

Nitric oxide (NO) produced by nitric oxide synthase (NOS) in human red blood cells (RBCs) was shown to depend on shear stress and to exhibit important biological functions, such as inhibition of platelet activation. In the present study we hypothesized that exercise-induced shear stress stimulates RBC-NOS activation pathways, NO signaling, and deformability of human RBCs.

**Methods/Findings:**

Fifteen male subjects conducted an exercise test with venous blood sampling before and after running on a treadmill for 1 hour. Immunohistochemical staining as well as western blot analysis were used to determine phosphorylation and thus activation of Akt kinase and RBC-NOS as well as accumulation of cyclic guanylyl monophosphate (cGMP) induced by the intervention. The data revealed that activation of NO upstream located enzyme Akt kinase was significantly increased after the test. Phosphorylation of RBC-NOSSer^1177^ was also significantly increased after exercise, indicating activation of RBC-NOS through Akt kinase. Total detectable RBC-NOS content and phosphorylation of RBC-NOSThr^495^ were not affected by the intervention. NO production by RBCs, determined by DAF fluorometry, and RBC deformability, measured via laser-assisted-optical-rotational red cell analyzer, were also significantly increased after the exercise test. The content of the NO downstream signaling molecule cGMP increased after the test. Pharmacological inhibition of phosphatidylinositol 3 (PI3)-kinase/Akt kinase pathway led to a decrease in RBC-NOS activation, NO production and RBC deformability.

**Conclusion/Significance:**

This human *in vivo* study first-time provides strong evidence that exercise-induced shear stress stimuli activate RBC-NOS via the PI3-kinase/Akt kinase pathway. Actively RBC-NOS-produced NO in human RBCs is critical to maintain RBC deformability. Our data gain insights into human RBC-NOS regulation by exercise and, therefore, will stimulate new therapeutic exercise-based approaches for patients with microvascular disorders.

## Introduction

Nitric oxide (NO), a highly reactive and short-lived diffusible molecule regulates central physiological mechanisms, e.g. vascular tone, macrophage-mediated neurotoxicity, anti-apoptotic activity [1], or mitogen-activated protein (MAP) kinase signaling [2]. Thus, depending on its cellular concentration, NO acts as a physiologcal messenger on the one side and displays cytotoxic activity on the other side (for review see [3]). NO is enzymatically produced through four major isoforms of nitric oxide synthases (NOS): calcium-independent inducible NOS (iNOS) [4], calcium-dependent and constitutively expressed neuronal NOS (nNOS), endothelial NOS (eNOS) [5], [6] and red blood cell NOS (RBC-NOS) [7], [8].

*In vitro* investigations have shown that pharmacological stimuli positively influence RBC-NOS activation [9]. Application of recombinant human erythropoietin (rhEPO) increased RBC-NOS activity through phosphorylation of its serine^1177^ (RBC-NOSSer^1177^)-residue that resulted in increased NO production. It was further shown that the phosphatidylinositol 3 (PI3)-kinase/Akt kinase pathway is involved in this process [9]. Additionally, NO produced by RBC-NOS has been found to regulate platelet activation and the deformability of RBC membranes [8]. Recent *in vitro* examinations have shown that the application of continuous shear stress also activates RBC-NOS and NO production [10]. This leads to the indication of important influences of mechanical stimulations in the vascular bed on RBC-NOS activation and subsequent NO formation to maintain RBCs deformability and, thus, peripheral oxygen supply.

Importantly, it is known that physical exercise results in increased peripheral shear stress in the vascular bed [11]. In this context, it was recently demonstrated in untrained rodents that physical exercise positively influences RBC deformability [12]. It can be speculated from these results that the improvement in RBC deformability is caused by RBC-NOS activation, because RBC-NOS represents an important source of NO production [8], [13], with NO released in RBCs both leading to improved deformability [14] and potentially positively influencing vascular regulation. But these very important direct correlations have not been investigated in detail, yet.

In contrast, our group demonstrated in a human *in vivo* investigation [15] that high-intensive exercise induces protein catabolism and thus negatively affects the RBC-NOS activity Similar effects were found in trained rodents where high intensity exercise impaired characteristics of RBCs’ plasma membrane resulting in diminished deformability [12], [16].

It can be stated that *in vitro*, but also possibly *in vivo,* shear stress induced e.g. by moderate exercise does not lead to protein catabolism and therefore improves RBCs rheological characteristics. However, the underlying *in vivo* mechanisms of these regulations are far from being resolved. Therefore, we hypothesized in the present study that acute moderate physical exercise stimulates and improves RBC-NOS activity, RBC NO production and deformability in humans. Additionally, we proved the questions whether NOS-regulating upstream located signaling pathways, specifically Akt kinase signaling [8], are involved in these important processes *in vivo*.

## Methods

### Ethical Approval

The protocols used in this study were approved by the ethics committee of the German Sports University Cologne. These protocols align with the Declaration of Helsinki and all participants gave written informed consent to participate in this study.

### Selection of Subjects

Fifteen male subjects participated in this study. Basal anthropometric parameters of the subjects were as follows (mean ± SD): age [years]: 27±3.6, height [cm]: 182±4.0, weight [kg]: 79±8.9. All subjects abstained from alcohol consumption for 24 hours prior to and during the training intervention and were non-smokers.

### Exercise Protocol and Blood Sampling of the *in vivo* Investigations

During the acute moderate running test (AMRT) the participants exercised at a velocity corresponding to 70% of their individual 4 mmol lactate threshold for 1 hour. This velocity was determined for each subject in a preliminary incremental running step test (IRT) which was performed 21 days prior AMRT. This three week interruption phase between the tests was chosen to avoid post-exercise plasma expansion, intensified haemolysis or disturbances in erythropoiesis [17] to interfere subsequent testing. The test persons were also advised to abandon high intensity exercise during the period of rest. The IRT started at a velocity of 2.0 m*sec^–1^ and the speed increased every 5 min by 0.5 m*sec^–1^ until subjective exhaustion. Every interval was followed by a 30 sec break to obtain 20 µl blood from the earlobe for lactate analysis. Capillary blood lactate concentrations were measured with EBIO plus (EKF Diagnostic Sales GmbH, Magdeburg, Germany). A calculation matrix developed at the German Sport University was used to determine the individual 4 mmol lactate threshold, the 70% of this threshold and the corresponding velocity applied during AMRT. The average running speed during AMRT was 2.39±0.41 m*sec^–1^.

Blood gas analysis of capillary blood was carried out (AVL Omni Series Blood Gas Analyzer, Roche Diagnostics, Mannheim, Germany) in order to obtain pH, partial pressure of arterial carbon dioxide (pCO_2_), partial pressure of oxygen that is dissolved in arterial blood (pO_2_), and arterial haemoglobin oxygen saturation (sO_2_) Pre AMRT and Post AMRT (Table 1).

**Table 1:** Arterial blood gas analysis Pre- and Post-AMRT.

*Parameter*	*Pre-AMRT*	Post-AMRT
pH	7.40±0.005	7.39±0.005 P>0.05
pCO_2_ (mmHg)	38.32±1.10	37.36±0.34 P>0.05
pO_2_ (mmHg)	78.18±1.43	79.14±0.88 P>0.05.
sO_2_ (%)	99.58±0.22	98.18±0.95 P>0.05.

Data are presented as mean ± S.E.M of n = 15.

Additional blood samples were taken from the subjects’ vena mediana cubiti Pre and Post AMRT to determine Akt kinase and RBC-NOS phosphorylation, NO production in RBCs and RBC deformability. To avoid any additional shear stress which may alter NO production in the RBCs, blood was collected without a tourniquet Pre and Post using the Safety-Multifly-Set (21G, 0,8×19 mm; Sarstedt, Nürnbrecht, Germany) connected to a 5 mL syringe. 4 mL blood was slowly drawn from the vein and immediately anticoagulated using either Heparin vacutainers (BD Vacutainer, Franklin Lakes, USA).

### *In vitro* Analysis of RBC-NOS Activation through PI3 Kinase Pathway

An additional *in vitro* approach was carried out to investigate the influence of wortmannin on RBC-NOS serine^1177^ phosphorylation (RBC-NOSSer^1177^), RBC NO production and RBC deformability. Wortmannin has been shown to inhibit PI3 kinase, thereby reducing Akt signaling. This reduction decreases phosphorylation of the eNOSSer^1177^ [18]. Wortmannin was used to determine a direct link between PI3 kinase/Akt kinase/RBC-NOS and NO signaling in human RBCs. NO production was determined Pre and Post AMRT via DAF fluorometry (see below). Post AMRT blood samples were additionally incubated with wortmannin (c = 10 µM) for 30 min at 37°C to investigate whether exercise-induced NO production decreases upon PI3 kinase inhibition. The concentration and incubation time for wortmannin experiments were preliminary tested.

Post AMRT additional blood was taken to also investigate phosphorylation of RBC-NOSSer^1177^ and RBC deformability depending on PI3 kinase inhibition. Parameters were determined before (0 min) and after (30 min) wortmannin incubation. Simultaneous sample incubation with phosphate buffered saline (Dulbeccós Phosphate-Buffered Saline (PBS) (1x), without calcium and magnesium, PAA, Paschin, Austria) adjusted to physiological pH 7.4 served as control.

### Immunohistochemical Procedure

Immunostaining of RBCs as described hereinafter has been applied in various studies [8], [10], [15], [19]. RBCs were immunostained and analyzed by semi-quantitative approach as described previously [15]. According to the protocol of Suhr et al. [15] venous blood from either the exercise intervention or from wortmannin incubation was fixed with 4% paraformaldehyde (v/v; 1/1) immediately after blood sampling. After repeated washing procedures RBCs were dispersed on a slide and heat fixed. The RBCs were washed in 0.1 M tris-buffered saline (TBS), permeabilized for 45 min in 0.1% trypsin, placed in a solution of 2% hydrogen peroxide and 80% methanol TBS for 20 min, and treated with 3% milk powder in 0.1 M for 30 min at room temperature. The test area of each slide was incubated with the respective primary antibody against eNOS (dilution 1∶1000, Biomol, Hamburg, Germany), eNOSSer^1177^, eNOSThr^495^ (dilution 1∶500, Upstate, Lake Placid, USA), cGMP (dilution 1∶3000, Chemicon/Millipore GmbH, Schwalbach, Germany) or phosphoAktSer^473^ (dilution 1∶500, Cell Signaling, Danvers, USA) for 1 hour. The control area was incubated in the absence of the first antibody. After repeated washing the sections were incubated with a secondary goat-anti-rabbit antibody (dilution 1∶400, Dako, Glostrup, Denmark) for 1 hour. A streptavidin-horseradish-peroxidase complex (Amersham, Buckinghamshire, England) was applied as a detection system (dilution 1∶150) for 30 min. The staining was developed using 3,3-diaminobenzidine-tetrahydrochloride solution (Sigma, St. Louis, USA) in 0.1 M TBS.

Following the protocol of Kleinbongard et al. [8] and Ludolph et al. [20] for intensity analysis of immunostaining in RBCs, the gray values (television densitometry) of 100 RBCs (test area) from at least four randomly selected pictures and 50 RBCs (control area) from at least two randomly selected pictures of each slide were measured. The intensity of immunostaining was reported as the mean of measuring RBC gray value minus background gray value. The background gray value was detected at a cell-free area of the slide. Finally, the gray values of the test area (minus background) and the control area (minus background) were subtracted. For staining intensity detection, a Leica microscope coupled to a CCD-camera (DXC-1850P, Sony, Germany) was used and the analysis was conducted using the software “Image J” (National Institutes of Health, Bethesda, Maryland, USA). Magnification for all images was 400-fold.

### Western Blot Analysis

Additional western blot analyses were performed to confirm the results obtained in the immunohistochemical procedure. For this purpose heparinized venous blood was taken Pre and Post AMRT. RBCs were separated at 800×g for 10 min at 4°C. RBC protein fractions were extracted using the ProteoExtract Subcellular Proteome Extraction Kit (CalBiochem, Darmstadt, Germany). The mild procedure yields proteins in their native state. 5×10^6^ RBCs were used for the extraction. RBCs were repeatedly washed (v/v; 1∶20; RBCs : washing buffer) at 800×g for 10 min at 4°C using supplied washing buffer. RBCs were then incubated with supplied extraction buffer for 10 min at 4°C by gentle shaking to release cytosolic proteins. After centrifugation at 1,000×g for 10 min at 4°C supernatant with cytosolic proteins were stored on ice until further processing. Subsequently, a second extraction buffer was added to the remaining pellet, incubated for 30 min at 4°C and the sample was centrifuged at 20,000×g for 10 min and 4°C. The supernatant with the solubilized membrane proteins was stored on ice. A third extraction buffer was added to the remaining pellet, incubated for 10 min at 4°C on a shaker and the sample was then centrifuged at 20,000×g for 10 min and 4°C. This supernatant is aimed to contain nucleic proteins and was discarded because mature RBCs are anucleate cells [21]. Finally, a fourth extraction buffer was added to the pellet to solubilize components of the cytoskeleton.

Protein concentration of all four fractions was determined using the DC-Protein Assay Kit (BioRad, Munich, Germany). A preliminary test revealed that the relevant proteins were present in the cytosolic fraction that therefore was used in subsequent western blot analysis. A total of 150 µg protein (fraction 1) was loaded fivefold into the lanes of a 4–12% Bis-Tris gel (BioRad, Munich, Germany) next to the Precision Plus Protein™ Standard (BioRad, Munich, Germany). Proteins were separated according to their charge and mass in a 1 x MOPS running buffer (BioRad, Munich, Germany) and transferred to a polyvinylidene fluoride membrane (0.45 µm pore size) and probed with antibodies against total eNOS (dilution 1∶800, Biomol, Hamburg, Germany), phosphorylated eNOSSer^1177^, phosphorylated eNOSThr^495^ (dilution 1∶800, Upstate, Lake Placid, USA), cGMP (dilution 1∶1:800, Chemicon/Millipore GmbH, Schwalbach, Germany) or phosphorylated AktSer^473^ (dilution 1∶1:800, Cell Signaling, Danvers, USA). The membrane was then incubated with a secondary goat-anti-rabbit peroxidise conjugated antibody (dilution 1∶2000, Thermo Scientific, Bonn, Germany). After development of immunoreactive bands by means of an enhanced chemiluminescence kit (Thermo Scientific, Darmstadt, Germany), relative band intensities were quantified using software “Image J”. The difference in intensity between Pre and Post exercise condition was calculated.

### DAF Fluorometry

As described by Kojima et al. [22] and Itoh et al. [23] the cell-permeable 4,5-diaminofluorescein diacetate (DAF-2DA) (Alexis Biochemicals, Lörrach, Germany) indicates NO-production inside cells, where DAF-2A is N-nitrosated and thereby forms the fluorescing triazolofluorescein (DAF-2T). DAF-2A does not react directly with the NO radical, but rather with nitrous anhydride (N_2_O_3_), which is formed by autoxidation of NO in air [22], [24], [25]. DAF-2DA was used to detect NO production in human RBCs before and after the AMRT intervention and after wortmannin application. Pre and Post AMRT, venous blood was equilibrated with DAF-2DA solution (c = 10 µM) for 3 min. After incubation of the blood with DAF-2DA, 50 µl of blood were pipetted and dispersed onto a little glass bottom petri dish. To investigate the question whether the fluorescent signal was NO- and NOS-specific, wortmannin (c = 10 µM) was added to another 100 µl blood sample (Post AMRT) and was incubated for 30 min. The whole blood was imaged on a confocal laser scanning microscope (LSM 510 Meta, Zeiss, Germany) with an Argon laser used to provide fluorescence excitation at 488 nm.

### RBC Deformability

Immediately after sampling, venous heparinized blood was mixed (v/v; blood/medium; 1∶250) with an isotonic viscous medium (0.14 mM Polyvinylpyrrolidone (PVP), osmolality 300 mOsmol*L^−1^, viscosity 32 mPa*s at 37°C; Mechatronics, Netherlands) for deformability measurements. All measurements were performed at physiological pH 7.4 and 37°C. Deformability was determined at various physiological and supra-physiological fluid shear stresses by laser diffraction analysis using the laser-assisted optical rotational red cell analyzer (LORRCA, RR Mechatronics, Netherlands). The LORRCA has been described in detail elsewhere [26], Briefly, 1 mL of the blood/PVP mixture was sheared in a Couette system. A laser beam was directed through the sheared sample and the diffraction pattern produced by the deformed red cells was analyzed. On the basis of the geometry of the elliptical diffraction pattern, an elongation index (EI) was calculated by the software as EI = (L-W)*(L+W)^–1^, where L and W represent length and width of the diffraction pattern, respectively. EI values were calculated for nine shear rates, in particular 0.3, 0.57, 1.08, 2.04, 3.87, 7.34, 13.92, 26.38 and 50 Pa. EI values were plotted as a function and according to Baskurt et al. [27], the Lineweaver-Burke equation was used to calculate the maximum deformability (EImax) and the shear stress required for one-half of this maximal deformation (SS ½). Increased SS ½ values indicate decreased RBC deformability.

Additionally, Post AMRT heparinized blood samples were also incubated with either PBS or wortmannin (c = 10 µM) for 30 min at 37°C to inhibit PI3 kinase. RBC deformability was measured after 0 and 30 min using the LORRCA as described previously.

### Statistical Analysis

Statistical analyses of the data were performed by using statistics software packages (STATISTICA for Windows, 7.0, Statsoft, Tulsa, USA and Origin 8.5 Pro, Northampton, USA). Data were analyzed by paired Student’s t-test to determine the significance of any differences between the Pre and Post situation. For comparison of wortmannin data one-way analysis of variances (ANOVA) was applied. Descriptive statistics of the data is presented as mean ± standard error of means (S.E.M.) unless described otherwise. Statistical differences were considered to be significant for values of *P*<0.05.

## Results

### Immunohistochemical Staining of Akt Kinase, RBC-NOS and cGMP

Immunohistochemical straining was performed Pre and Post AMRT to investigate whether moderate exercise influences RBC-NOS activation pathway.

**Figure 1:**
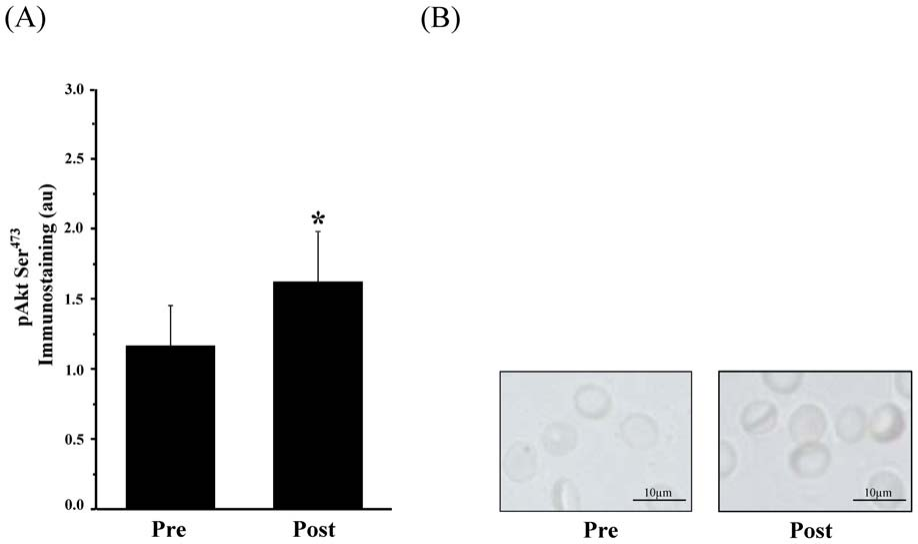
Change in phosphoAktSer^473^ before (Pre) and after (Post) AMRT determined by immunohistochemistry. (A) Bars show statistical analysis of gray values (au) between Pre and Post condition. The phosphoAktSer^473^ signal in human RBCs significantly increased from Pre to Post (*P*<0.05). (B) Pictures show representative phosphoAktSer^473^ staining before and after the test, respectively. Magnification for all images was 400-fold. Data in (A) are presented as mean ± S.E.M of n = 6.

*AktSer^473^*. To determine the impact of the intervention on Akt kinase activation, phosphorylation of Akt kinase on its Ser^473^ residue was examined before and after the test. The statistical analysis of phosphoAktSer^473^ revealed that the value significantly increased from 1.16±0.29 au Pre exercise to 1.61±0.39 au Post exercise (*P*<0.05, Figure 1 A, B).

#### RBC-NOS and its activation sites

**Figure 2:**
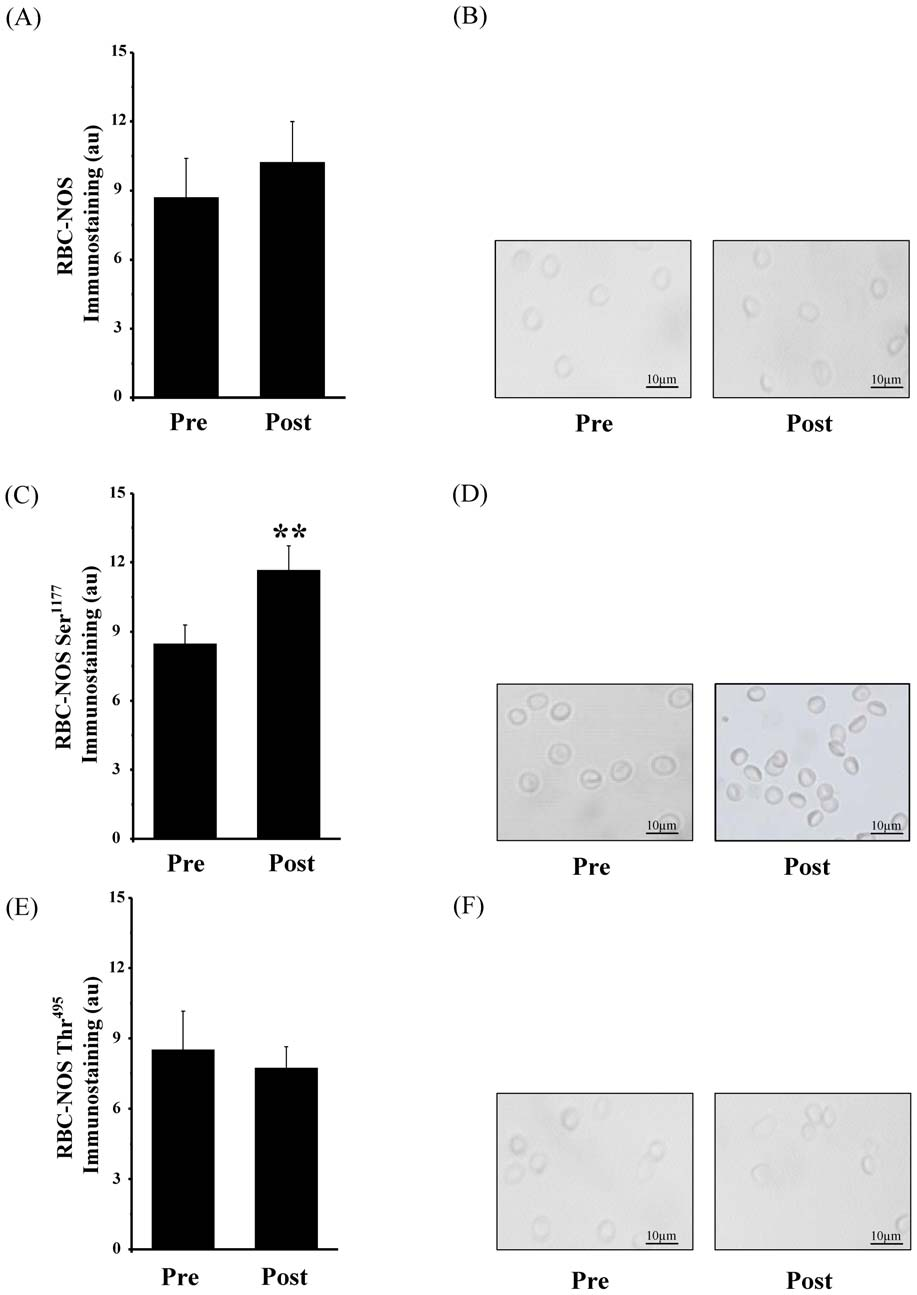
Change in detectable RBC-NOS and RBC-NOS phosphorylation before (Pre) and after (Post) AMRT determined by immunohistochemistry. (A) Bars show statistical analysis of gray values (au) revealing no statistical difference in detectable total RBC-NOS between Pre and Post conditions. (B) shows representative detectable RBC-NOS staining before and after the test, respectively. (C) The data reveal that RBC-NOS phosphorylation at Ser^1177^ residue significantly increased from the Pre to Post situation (*P*<0.01). (D) Pictures show representative RBC-NOSSer^1177^ staining before and after the test, respectively. (E) The graph shows no statistical difference in gray values (au) between Pre and Post conditions for RBC-NOSThr^495^. (F) Pictures show representative RBC-NOSThr^495^ staining before and after the test, respectively. Magnification for all images was 400-fold. Data (A), (C) and (E) are presented as mean ± S.E.M of n = 6.

The total detectable RBC-NOS, RBC-NOSSer^1177^ and RBC-NOSThr^495^ signal intensity were examined and the statistical evaluation revealed no difference in total detectable RBC-NOS between the Pre-situation and the Post-situation (Pre: 8.69±1.69 arbitrary units (au) vs. Post: 10.22±1.78 au, *P*>0.05, Figure 2 A, B). The statistical analysis of gray values of subjects’ RBCs against phosphorylated RBC-NOSSer^1177^ showed significantly increased levels Post exercise compared to Pre exercise (Pre: 8.47±0.82 au vs. Post: 11.65±1.07 au, *P*<0.01, Figure 2 C, D). RBC-NOSThr^495^ staining showed no significant difference between the Pre (8.52±1.65 au) and Post situation (7.73±0.93 au, *P*>0.05, Figure 2 E, F).

**Figure 3:**
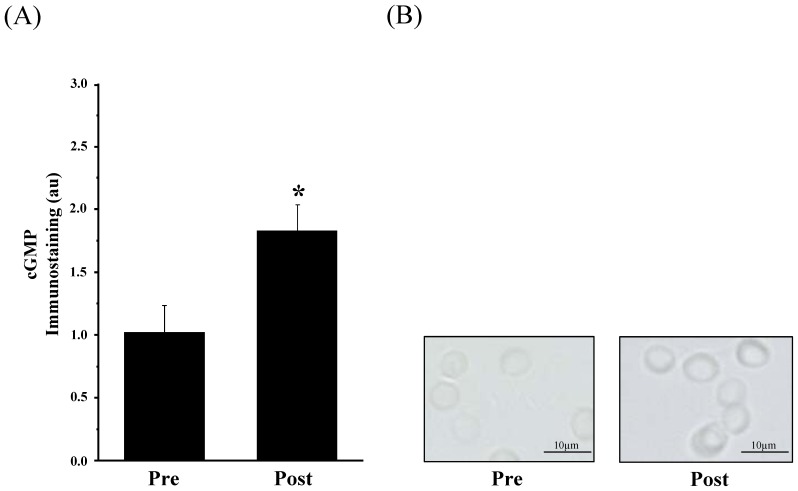
Change in cGMP before (Pre) and after (Post) AMRT determined by immunohistochemistry. (A) Bars show statistical analysis of gray values (au) between Pre and Post conditions. The cGMP signal in human RBCs significantly increased at the end of the test (Post) (*P*<0.05). (B) Pictures show representative cGMP stainings before and after the test, respectively. Magnification for all images was 400-fold. Data in (A) are presented as mean ± S.E.M of n = 6.

*cGMP*. To further verify possible NO formation by the activated RBC-NOSSer^1177^ we investigated downstream target proteins of NO. It is well known that cyclic guanosine monophosphate (cGMP) is a prominent target of NO in endothelial cells [28], [29] and RBCs [30], [31]. Therefore, we conducted evaluations of cGMP alterations after the applied exercise stimulation. The statistical analysis of the cGMP signal revealed significantly increased signal intensities from 1.02±0.21 au Pre exercise to 1.80±0.13 au Post exercise (*P*<0.05, Figures 3 A, B).

#### In vitro experiments

**Figure 4:**
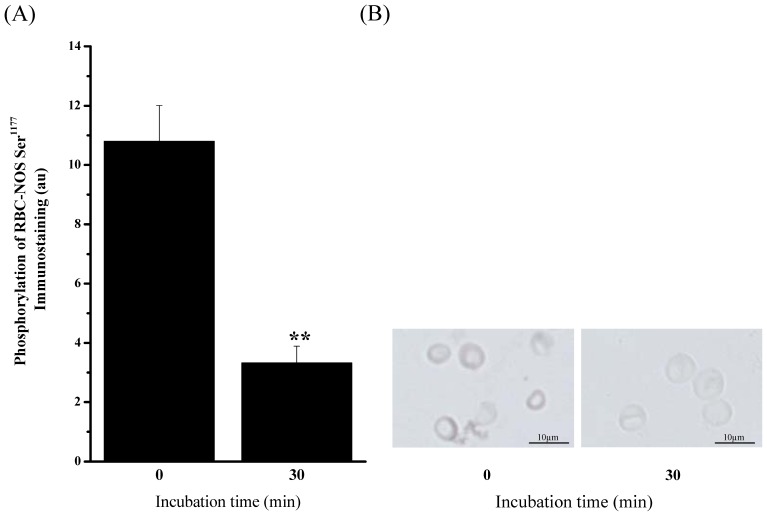
Change in RBC-NOSSer^1177^ after PI3 kinase inhibition via wortmannin determined by immunohistochemistry. (A) Bars show statistical analysis of gray values (au) before (0 min) and after a 30 min incubation of whole blood with PI3 kinase inhibitor wortmannin (c = 10 µM). RBC-NOSSer^1177^-signal significantly decreased after wortmannin incubation (*P*<0.01). (B) Pictures show representative RBC-NOSSer^1177^ staining before and after the test, respectively. Data in (A) is presented as mean ± S.E.M of n = 6.

The *ex-vivo* results revealed that moderate exercise activates both Akt kinase and RBC-NOS. We thus performed *in vitro* experiments to verify the observed *in vivo* results. The conducted *in vitro* approach investigated RBC-NOS activation via the PI3 kinase/Akt kinase pathway. For this purpose phosphorylation of RBC-NOS at Ser^1177^ was investigated after PI3 kinase inhibition using wortmannin [25]. The statistical analysis revealed that the analyzed signal intensity significantly decreased from 10.80±1.20 au Pre incubation (0 min) to 3.30±0.56 au Post incubation (30 min) (*P*<0.01, Figures 4 A, B).

### Western Blot Analysis of Akt Kinase, RBC-NOS and cGMP

Western Blot procedure served as an additional method, beside immunohistochemical approaches, to investigate whether moderate exercise influences RBC-NOS activation pathway and was performed Pre and Post AMRT. For the determination of relative intensity all obtained Pre-values were set to “1”. The corresponding Post-values were then calculated as the quotient of the Pre-values.

**Figure 5:**
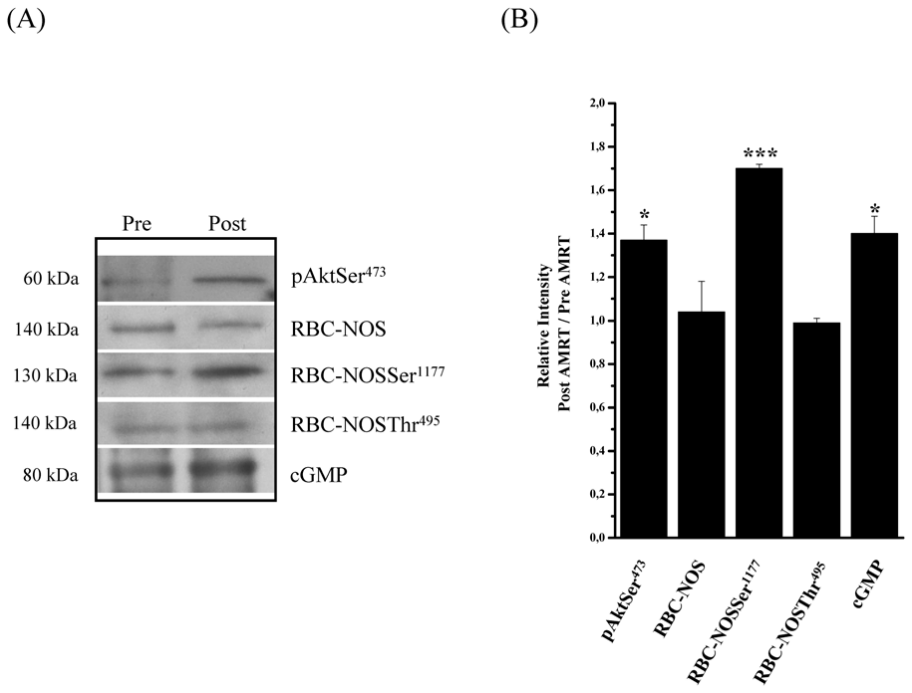
Change in phosphoAktSer^473^, RBC-NOS and cGMP before (Pre) and after (Post) AMRT determined by western blot analysis. (A) Specific representative bands for phosphoAktSer^473^, total detectable RBC-NOS, RBC-NOSSer^1177^, RBC-NOSThr^495^ and cGMP migrated at 60kDa, 140kDa, 130kDa, 140kDa and 80kDa, respectively. PhosphoAktSer^473^, RBC-NOSSer^1177^ and cGMP obtained Post-AMRT were significantly increased compared to Pre-AMRT. Bands obtained from total detectable RBC-NOS and RBC-NOSThr^495^ remained unchanged. (B) Relative intensity obtained from western blot bands was significantly increased for phosphoAktSer^473^ (*P*<0.05), RBC-NOSSer^1177^ (*P*<0.001) and cGMP (*P*<0.05) Post-AMRT in relation to Pre- AMRT (set to “1”). Relative intensity of total detectable RBC-NOS and RBC-NOSThr^495^ remained unaltered. Data in (B) are presented as mean ± S.E.M of n = 6.

Western blot analysis revealed that Post-AMRT the investigated proteins, phosphoAktSer^473^ (Post: 1.37±0.07, *P*<0.05), RBC-NOSSer^1177^ (Post: 1.7±0.02, *P*<0.001), and cGMP (1.4±0.08, *P*<0.05), were significantly increased compared to Pre-AMRT (Figure 5A, B). In contrast, total detectable RBC-NOS (1.04±0.14, *P*>0.05) and RBC-NOSThr^495^ (0.99±0.02, *P*>0.05) remained unchanged (Figure 5A, B).

### NO Production in RBCs

**Figure 6:**
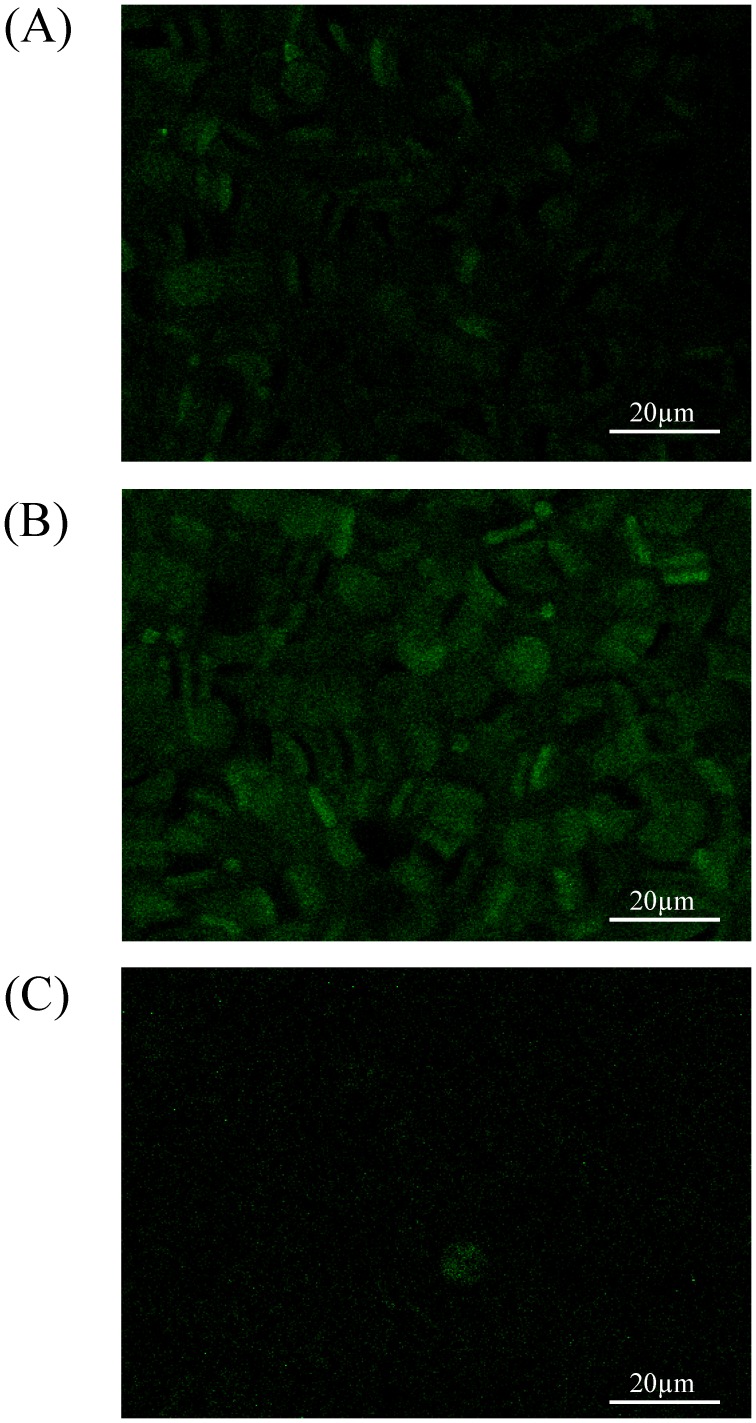
NO-generation in human RBCs after exercise. (A) DAF fluorometry revealed no detectable NO-generation Pre AMRT. (B) Strong NO generation signals were observed Post AMRT. (C) The NO generation was strongly reduced after AMRT by the PI3 kinase specific inhibitor wortmannin (c = 10 µM). Bar, 20 µm.

Due to an increased level of RBC-NOS phosphorylation at the Ser^1177^ residue after the AMRT intervention, the NO production in RBCs was investigated as a direct readout for RBC-NOSSer^1177^ activation. DAF-2DA experiments were conducted before (Pre) and directly after the AMRT intervention (Post). Figure 6A shows that almost no nitric oxide production was detected in human RBCs by DAF-2DA imaging Pre AMRT. In contrast, the NO production in human RBCs increased after the physical activation, as measured post-exercise (Figure 6B) and depicted by the green fluorescent signal.

The addition of the indirect RBC-NOS inhibitor wortmannin Post AMRT caused a down-regulation in NO production in human RBCs (Figure 6C).

### RBC Deformability Determined after AMRT

**Figure 7:**
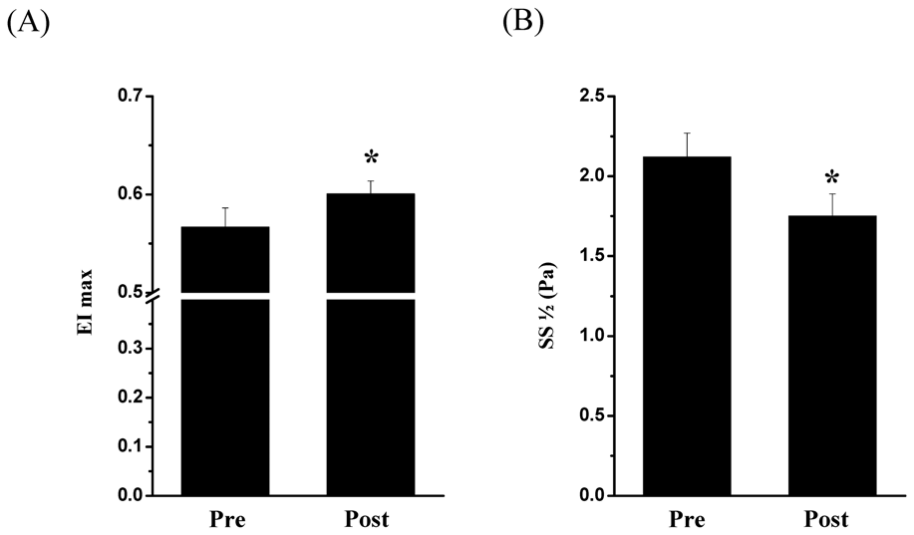
Influence of AMRT on RBC deformability. RBC deformability was obtained before (Pre) and after (Post) AMRT. (A) EImax significantly increased from Pre to Post exercise (*P*<0.05). (C) SS ½ significantly decreased Post AMRT. Data are presented as mean ± S.E.M of n = 15.

RBC deformability is crucial for the passage of RBCs through the microvasculature bed [32], [33] to ensure the oxygen supply to working tissues. The results obtained in this study showed that the RBC deformability was significantly influenced by the intervention. EI max values increased from 0.57±0.019 (Pre) to 0.6±0.013 (Post, *P*<0.05, Figure 7A). SS ½ significantly decreased from 2.12±0.158 Pa (Pre) to 1.75±0.14 Pa (Post, *P*<0.05, Figure 7B).

### *In vitro* Analysis of RBC Deformability in Dependence of PI3 Kinase Inhibition

**Figure 8:**
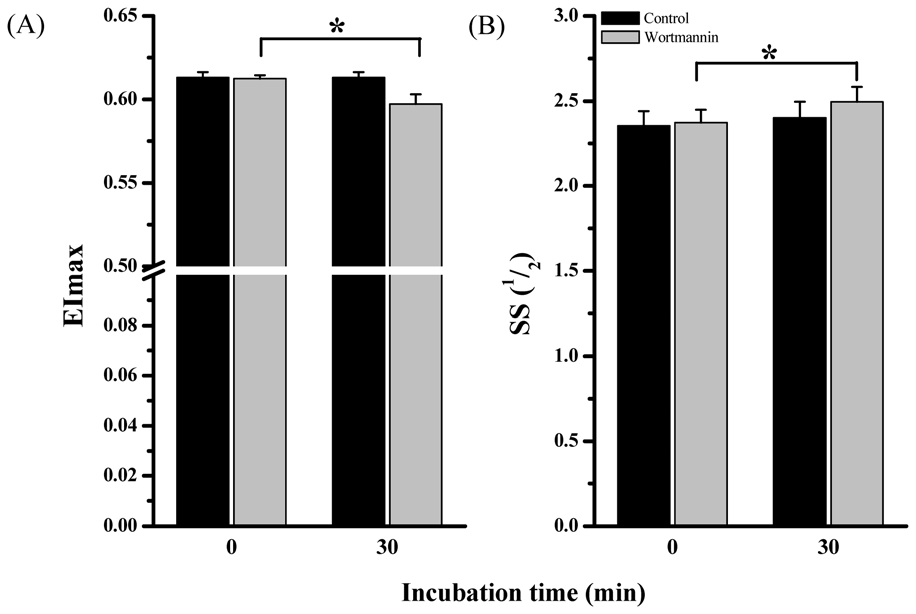
Influence of wortmannin on RBC deformability. RBC deformability was measured before and after 30 min incubation with wortmannin and PBS, as control. (A) No difference in EImax was observed after PBS treatment. After wortmannin incubation EImax was significantly decreased (*P*<0.05). (B) SS ½ was unchanged after PBS treatment but significantly increased after wortmannin application, indicating decreased deformability (*P*<0.05) Data are presented as mean ± S.E.M of n = 10.

Maximal RBC deformability of *in vitro* wortmannin-treated samples significantly decreased from 0.61±0.002 (0 min) to 0.59±0.006 (30 min incubation; *P*<0.05). EI max of control samples was determined to be 0.61±0.003 (0 min incubation) and 0.61±0.003 after the 30 min incubation (*P*>0.05; Figure 8A). In wortmannin-treated samples, SS ½ significantly increased from 2.37±0.08 (0 min) to 2.50±0.08 (30 min incubation; *P*<0.05). SS ½ of control samples was 2.36±0.09 (0 min) and 2.40±0.09 after the 30 min incubation (*P*>0.05; Figure 8B).

## Discussion

NO generation and –mediated signaling are highly important for the regulation of the peripheral vascular bed [28], [29] and NO produced by eNOS in endothelial cells plays a prominent role in these regulations (for review see [34], [35]). However, very recently the NOS protein localized in RBCs came into focus as an additional important NO generating source [8].

Although it has been shown *in vitro* that defined shear stresses regulate and manipulate the activation of RBC-NOS with subsequent NO production [10] and that intracellular NO generation is involved in the improvement of RBCs rheological characteristics [8], [14], it remains to be elucidated what molecular effects are promoted *in vivo* by moderate physical exercise on RBC regulation, specifically on RBC-NOS activation, NO generation, and subsequently on RBCs rheological characteristics.

In the present study, we therefore studied the *in vivo* effect of reinforced shear stress, caused by moderate exercise, on RBC-NOS protein, its activations sites, and –mediated NO signaling to outline that moderate physical exercise exerts a positive influence on the regulation of this biologically important enzyme.

The phosphorylation of RBC-NOSSer^1177^, indicating increased enzyme activity [8], [19], was significantly increased after AMRT, whereas total RBC-NOS and the phosphorylation of RBC-NOS at its Thr^495^ residue, indicating inhibition of NOS enzyme activity [36], were not affected by the applied exercise intervention. In line with RBC-NOS activation, the NO production was clearly increased after ARMT. These data indicate a beneficial effect of moderate exercise on RBC-NOS activity and subsequent NO production.

Next, signaling pathways that contribute to the activation of the RBC-NOS under exercise conditions were aimed to be identified. In endothelial cells eNOSSer^1177^ phosphorylation and thus eNOS activation depends on the phosphorylation of Akt kinase at its Ser^473^ through PI3 kinase [37], [38], [39]. Here, we report that *in vivo* phosphorylation of RBCs Akt kinase at Ser^473^ significantly increased after AMRT, indicating a direct molecular interaction between phosphorylation of Akt- and RBC-NOS activation. These data complement the study of Mihov and colleagues who showed, using an *in vitro* assay, that pharmacological stimulations increase Akt kinase activity through PI3 kinase activation in RBCs [9].

NO, as an unstable radical, reacts rapidly within the cell [1]. Different possible reaction routes of NO might include the oxidation to nitrite and nitrate or the binding to hemoglobin or soluble guanylylcylase (sGC) with subsequent increase of cGMP content. It is also discussed that NO possesses the bioactive potential to regulate functions of the RBC itself, e.g. deformability [13], [31]. In the present study we revealed that both, cGMP content and RBC deformability were significantly increased after AMRT by the described molecular routes. In the context of the present study it appears significant that RBC deformability increases upon exercise stimulation as RBCs exhibit an average diameter of about 7.5 µm [40], which is thus smaller than the diameter of the smallest capillaries having a diameter of 4.5 µm [40]. The RBCs transit through the capillaries to supply peripheral skeletal muscle and also cardiac areas with oxygen. Increased deformability thus allows the cells to respond to the changing demands by easing the passage through the vessel thereby increasing the oxygen supply during physical exercise. The interactions between membrane, peripheral and cytoskeleton proteins are responsible for the maintenance of RBC deformability and some of these interactions are modulated by protein kinase activity. It has been shown that deformability is significantly decreased by inhibition of PKC [41], a kinase that might also interact with NOS [42]. Also, it has been shown that RBCs contain large amounts of adenosine triphosphate (ATP) [43], a substance activating endothelial P_2y_ purinergic receptors, thereby resulting in synthesis of endothelial NO [44], [45] that leads to relaxation of vascular smooth muscles [46]. RBCs do not release ATP spontaneously, but upon mechanical deformation with the degree of deformation positively correlating with the ATP released [43]. This finding was supported by the a study of Wan and colleagues who showed that retraction of the spectrin-actin cytoskeleton network triggers the mechanosensitive ATP release [47]. Therefore, deformability of RBCs represents an important mechanism for the passage of RBCs through the microcirculation as well as for the control of vascular calibre. Patients with cystic fibrosis (CF) [48], primary pulmonary hypertension (PPH) [49] or diabetes [50] show less deformable RBCs and do not release ATP in response to mechanical deformation. These patients show also impaired endothelial NO synthesis [51], [52]. From the data we represent here it can be assumed that also NO synthesis in RBCs might be impaired in these patients, which might cause reduced RBC deformability, reduced ATP release and consequently impaired NO synthesis in the endothelium.

The function of cGMP in RBCs is only speculative, but it is conceivable that RBC -cGMP also influences vascular tonus possibly by activating potassium channels that control membrane potentials in response to altered intracellular calcium [53], [54]. However, further studies are needed to investigate this hypothesis.

Our data on human RBC-NOS modulation are the first to add important and molecular knowledge to the question whether exercise-induced increased shear stress promotes the activation of Akt kinase and RBC-NOS in RBCs thereby initiating beneficial alterations of RBC rheological properties.

To verify and support our obtained human *in vivo* results on RBC-NOS-related signaling, we conducted additional *in vitro* experiments by applying the PI3 kinase/Akt kinase inhibitor wortmannin [18] on human RBCs. We observed that the inhibition of PI3/Akt kinase pathway led to a decrease in RBC-NOSSer^1177^ phosphorylation. In line with these observations, NO production and RBC deformability were significantly decreased indicating a causal relation between RBC-NOS activation and RBC deformability that depends on PI3/Akt kinase signaling.

The novel key findings of this study are that moderate physical exercise is a sufficient stimulator for the activation of RBC-NOS directly resulting in improved RBC rheological properties. The molecular mechanisms are highly similar to those observed in endothelial cells. Exercise-induced shear stress results in activation of the PI3 kinase/Akt kinase pathway that in turn activates RBC-NOS by phosphorylation at Ser^1177^. Activated RBC-NOS generates NO in RBCs that is mandatory to beneficially promote RBC rheological functions, e.g. deformability.

Therefore, the presented data gain important insights into the molecular pathways underlying RBC deformability. This knowledge is of great interest, because nowadays physical exercise programs are fundamental for the treatment of patients suffering from cardiac and cardiovascular diseases who may benefit from improved rheological properties. Further elucidations are required to investigate the effect of chronic exercise interventions on the discussed molecular RBC-NOS activation pathways and resulting biological functionality of RBC-NO- produced NO.
